# Long Term Surgical Outcome and Prognostic Factors of Atypical and Malignant Meningiomas

**DOI:** 10.1038/srep35743

**Published:** 2016-10-20

**Authors:** Yu-Chi Wang, Chi-Cheng Chuang, Kuo-Chen Wei, Cheng-Nen Chang, Shih-Tseng Lee, Chieh-Tsai Wu, Yung-Hsin Hsu, Tzu-Kan Lin, Peng-Wei Hsu, Yin-Cheng Huang, Chen-Kan Tseng, Chun-Chieh Wang, Yao-Liang Chen, Pin-Yuan Chen

**Affiliations:** 1 Department of Neurosurgery, Chang Gung Memorial Hospital at Linkou; School of Medicine, Chang Gung University, Taoyuan, Taiwan, ROC; 2 Department of Radiation Oncology, Chang Gung Memorial Hospital at Linkou, Chang Gung University, Taoyuan, Taiwan, ROC; 3 Department of Radiology, Chang Gung Memorial Hospital at Linkou, Chang Gung University, Taoyuan, Taiwan, ROC

## Abstract

Atypical and malignant meningiomas are rare. Our aim was to examine the treatment outcomes following surgical resection, and analyze associations between clinical characteristics and overall survival (OS) or relapse free survival (RFS). 102 patients with atypical or malignant meningiomas underwent microsurgical resection between June 2001 and November 2009 were analyzed retrospectively. We compared demographics, clinical characteristics, treatment, and complications. The five-year and ten-year overall survival rates were 93.5% and 83.4%, respectively. Three factors significantly reduced OS: Malignant meningiomas (p < 0.001), which also decreased RFS (p < 0.001); female patients (p = 0.049), and patients with Karnofsky Performance Status (KPS) < 70 at diagnosis (p = 0.009). Fifty two patients (51%) experienced tumor relapse. Total resection of tumors significantly impacted RFS (p = 0.013). Tumors located at parasagittal and posterior fossa area lead to higher relapse rate (p = 0.004). Subtotal resection without adjuvant radiotherapy lead to the worst local control of tumor (p = 0.030). An MIB-1 index <8% improved OS and RFS (p = 0.003). Total resection of atypical and malignant meningiomas provided better outcome and local control. Adjuvant radiation therapy is indicated for patients with malignant meningiomas, with incompletely excised tumors; or with tumors in the parasagittal or posterior fossa area. The MIB-1 index of the tumor is an independent prognostic factor of clinical outcome.

Meningiomas, one of the most common intracranial tumors, accounts for 13% to 26% of brain tumors and have an annual incidence of 6 per 100,000 people^1^. In 2000 and 2007, World Health Organization(WHO) graded meningiomas with histological standards. The standards are based on both of subjective and objective (mitotic index) criteria^2^: around 90% are benign (WHO grade I), 5–7% are atypical (WHO grade II), and only 1–3% are considered anaplastic or malignant (WHO grade III)^3^. This recent adoption of modern grading criteria and the rarity of the malignant subtype have limited the amount of available data on the clinical behavior, outcomes, and optimal treatment of meningiomas^4^.

Benign meningiomas contribute to relatively good prognosis, but atypical and malignant meningiomas show rapid progression and more invasion. Five-year progression- free survival and overall survival of approximately 50% and 60%, respectively was shown^1^,^4^,^5^,^6^. Gross total resection (GTR) is the optimal surgical principle for benign meningiomas, but the standard of care for atypical and malignant meningiomas has yet to be established^7^,^8^. The two high grade histopathology tumor types are more brain invasive, and radical surgical strategies can lead to high morbidity and poor overall outcome^9^. Previous literature shows that age less than 40 years, cranial base meningiomas, and male sex are associated with recurrence in benign meningiomas that have been subtotally excised, but such data are not available for atypical and anaplastic meningiomas^1^,^10^.

Both benign and malignant meningiomas have an equal propensity to bleed, whereas malignant tumors are not necessarily more vascular than benign tumors^1^. Pre-operative embolization of meningiomas helps reduce intra-operative tumor bleeding, which may decrease surgical time and make surgical resection easier^11^. The practice patterns of embolization prior to surgery vary widely^12^,^13^, and not all neurosurgeons consider embolization due to possible complications^14^.

It remains controversial about the role of adjuvant radiotherapy and stereotatic radiosurgery after surgical resection^15^,^16^,^17^,^18^,^19^. There is evidence supporting early addition of adjuvant radiotherapy after GTR to achieve better local tumor control^15^,^20^, but this is not universally recommended^21^,^22^.

To establish optimal treatment strategies, accurate evaluation of the prognostic factors in atypical and malignant meningiomas is essential. To define the correlation factor with atypical and malignant meningioma progression or recurrence, we retrospectively analyzed the clinical parameters and post-operative outcomes of patients with these tumors.

## Results

### Patient Demographics and Tumor Characteristics

The group (45 male and 57 female patients) had a KPS of 74.31 ± 8.27 at initial evaluation and a mean age of 57 ± 16.7 years during surgery (Table 1). According to histopathological grade with 2007 criteria, the menigniomas were stratified into two groups: WHO grade II (n = 86) and WHO grade III (n = 16). Tumors were also divided into four categories according to the anatomic location: convexity (n = 33); parasagittal (n = 32); skull base (n = 29); and posterior fossa (n = 8). The MIB-1 proliferative index that measures Ki-67 expression was available for 87 tumor specimens and had an average score of 8.6% ± 6.1%.

Most patients (90/102; 88.2%) survived until the end of follow up: mean survival time was 160 ± 8.9 months (range 13–192 months). The five-year overall survival rate was 93.5% (95% confidence interval [CI], 90.9–96.1%) and ten-year OS rate was 83.4% (95%CI, 78.1–88.7%). Twelve patients didn’t survive at the endpoint. One died due to lung cancer. For other 11 patients, the cause of death directly or indirectly associated with progressive meningioma, which included brain stem compression in 5 patients, pneumonia and respiratory failure in 4 patients, and severe sepsis in 2 patients.

**Figure 1:**
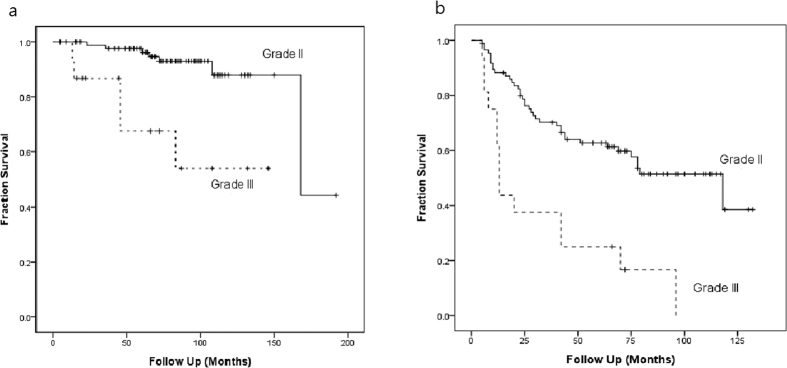
Kaplan-Meier estimates of overall survival (**a**) and relapse free survival (**b**) for patients with WHO grade II and grade III meningiomas. (p < 0.001).

Univariate analysis indicated that the overall survival was significantly influenced by sex, KPS, WHO grade, and MIB-1 index. Multivariate analysis indicated that sex, KPS, and WHO grade remained significant prognostic factors (Table 2). Kaplan-Meier analysis illustrated that malignant tumors (HR = 7.6, p < 0.001, Fig. 1a), female patients (HR = 9.3, p = 0.009) and KPS < 70 (HR = 5.1, p = 0.009) contribute to decreased OS. The location or size of tumors, the extent of resection, and adjuvant radiotherapy did not influence the overall survival significantly.

**Figure 2:**
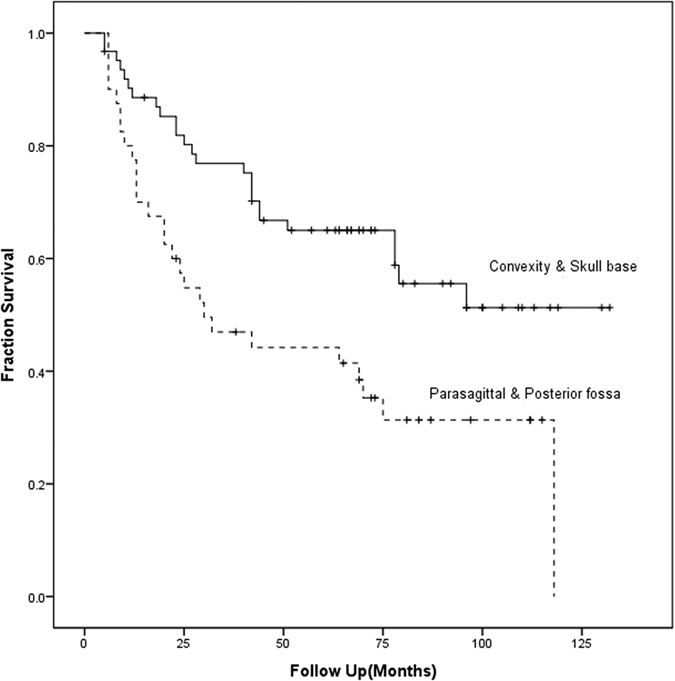
Relapse free survival between different location of tumors (p = 0.004).

**Figure 3:**
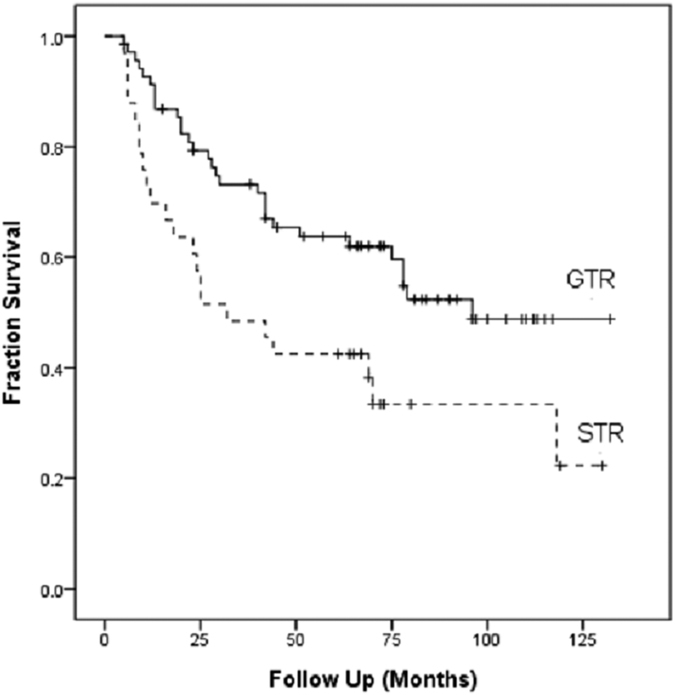
Kaplan-Meier analysis of relapse free survival as stratified by extent of resection. (p = 0.013) Abbreviations: GTR, gross total resection; STR, subtotal resection.

The disease relapsed during follow-up in 52 patients (51%). The average time to tumor relapse was 84.1 months (95% CI 77.3–90.9 months). The relapse free survival (RFS) rate at one, three and five years was 84.3%, 65.7% and 57.8%, respectively. Univariate analysis indicated that RFS was significantly influenced by WHO grade, tumor location, surgical extent, and MIB-1 index. Multivariate analysis indicated only WHO grade, surgical extent, and MIB-1 index remained significant factors (Table 2). No association was detected between adjuvant radiotherapy and duration of RFS. Tumor sizes were not associated with the completeness of tumor resection (p = 0.838) nor disease relapse (p = 0.883). Data analysis with Kaplan-Meier method made three inferences. First, a higher WHO grade of meningiomas was associated with a poorer RFS (HR = 3.5, p < 0.001, Fig. 1b). Second, tumor location affected prognosis: patients with tumors at convexity and skull base had better outcome, but patients with parasagittal and posterior fossa meningiomas experienced higher tumor relapse rate (HR = 2.2, p = 0.004, Fig. 2) Third, a comparison between GTR and STR revealed longer RFS occurred after complete resections (HR = 1.987, p = 0.013, Fig. 3).

We next divided patients into four subgroups to assess whether adjuvant radiotherapy in combination with surgical resection provided a RFS benefit for atypical and malignant meningioma patients: (1) GTR with adjuvant RT, (2) GTR without adjuvant RT, (3) STR with adjuvant RT, and (4) STR without adjuvant RT. Analysis showed a trend that patients who underwent GTR and RT had the lowest recurrent rate (41.8%), followed by those with GTR and without RT (53.6%), STR with RT (60.9%), while STR without RT had the highest recurrent rate (72.7%). Although patient groups who underwent GTR with versus without adjuvant RT did not show a significant difference by Kaplan-Meier survival analysis (p = 0.211), the patient groups who underwent STR showed a trend that adjuvant radiotherapy subgroup had better RFS (p = 0.053). The patient subgroup who received STR without RT had significantly shorter RFS than the other three subgroups (HR = 2.9, p = 0.008).

The tumor of 29 patients (28.4%) was embolized before surgery, and embolization induced no complications nor neurologic deficit. The mean size of tumors with embolization was 5.2 ± 1.9 cm, while the mean size of tumors without embolization was 5.3 ± 2.1 cm (*p* = 0.951). Embolization group had average intra-operative blood loss of 706 ± 217cc. Patients who didn’t receive embolization had average blood loss of 1043 ± 303cc. (p = 0.061). In addition, 23 of 29 patients with tumor embolization underwent GTR to tumors, which induced benefit to better RFS significantly. In other 73 patients not to have embolization, 46 had tumor total resection. The total resection rate was relatively higher in the patients receiving pre-operation embolization (79.3% vs 63.0%, *p* = 0.086, Table 3).

**Figure 4:**
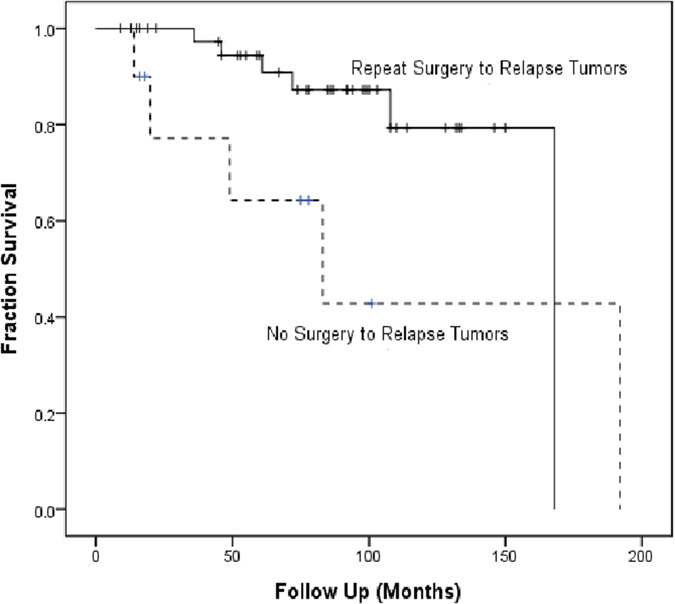
Kaplan-Meier analysis to overall survival of patients with relapse tumors. Repeat surgery associated with a better overall survival (p = 0.066).

Forty-five of 52 patients (86.5%) who developed tumor relapse during follow up received salvage therapy. Thirty-two patients (61.5%) underwent treatment with repeat surgery and radiotherapy; 10 patients (19.2%) underwent repeat surgery alone; and 3 patients (5.8%) underwent radiotherapy or radiosurgery alone. Factors associated with the decision for the mode of salvage therapy were not uniformly available. Of the 42 patients undergoing second operation to relapse tumors, 36 survived at the end. On the other hand, 5 of 10 patients without repeat surgical treatment (no salvage treatment or radiotherapy/radiosurgery alone) survived at the end. Survival rate were 85.7% and 50%, respectively (*p* = 0.025). Kaplan-Meier analysis also demonstrated a survival benefit with repeat operation to relapse disease.(p = 0.066, Fig. 4).

### Rate of complications

No deaths occurred during or following microsurgical tumor resection, within 30 days. Twenty five patients (24.5%) experienced at least one surgical, medical or neurological complication after their surgery. Three patients (2.9%) developed cardiac complications and pneumonia. Surgical complications (n = 16, 15.8%) included 6 wound infections or breakdown, 5 post-operative hematoma requiring evacuation, 3 cerebrospinal fluid leakage, and 2 hydrocephalus. On the other hand, 10 patients experienced significant neurological morbidity (9.8%) related to their tumor resection, such as cranial neuropathy and hemiparesis. Some patients had self-limiting symptoms like dizziness, headache, and skin irritation after radiotherapy, but there were no severe acute side effects.

## Discussion

Atypical and malignant meningioma patients comprised 4.2% and 1.2% of the meningioma cohort in the USA^23^, similar to the meningioma cohort (n = 936) in Finland^24^: grade I 94.3%, grade II 4.7%, and grade III 1.0%^24^. In our study, 922 patients had newly diagnosed meningiomas at our institute, and patients with WHO grade II and III meningiomas accounted for 9.2% (n = 86) and 1.7% (n = 16), respectively. Kshettry *et al*. also reported that Black and Asian Pacific Islander races were both associated with the highest incidence of WHO II/III meningiomas^23^. To the best of our knowledge, the present study is one of the largest case series that examined the treatment outcomes for meningiomas with atypical and malignant histology of Asian populations.

In the current study, the five-year overall survival rate of the patients with grade II and grade III meningioma was 97.5% and 67.4%, while median survival time was 167 months and 72 months, respectively. Perr *et al*. reported that the median survival time for patients with grade II lesions ranged from 10 to 14 years and they showed 75% 5-year overall survival rate. The median survival time for patients with grade III lesions was 1.5 years, with a 5-year survival rate of 32%^25^. A review article reported a median 5-year overall survival of 67.5% (range 51–100%) in grade II patients and 55.6% (range 27–80.8%) in grade III patients^4^. The relative higher survival rate in the present cohort is probably because of the aggressive retreatment strategy, especially surgery for recurrent tumors. Most patients with tumor recurrence or progression (49/52; 94%) underwent salvage treatment (surgery, radiotherapy, or both). We performed 80 repeat surgeries in these patients, and one patient received eight surgical resections.

In the current series, patient sex was a prognostic factor to overall survival, since female patients had significantly poorer outcome than male patients (HR: 9.3, p = 0.009). In our study, patient age at diagnosis had no influence on overall survival. In contrast, D. Pasquier *et al*. indicated that an age of >60 years was a significant adverse prognostic factor, whereas sex did not correlate with overall survival^26^. Milosevic *et al*.^27^. reported that an age of less than 40 years was associated with a favorable outcome. In our opinion, patient’s physical condition, as aptly measured by KPS, is more important than age. Our finding of KPS as a prognostic factor supports this explanation: patients with KPS ≥ 70 had better overall survival than those with KPS < 70 (HR = 5.1, p = 0.009).

Because several classification criteria exist for grading histopathology severity of meningiomas in the past two decades, evaluating reported treatment results for these rare tumors has been difficult. In our series, we adapted the latest 2007 WHO revision classification scheme and showed that the WHO grade of meningioma definitely plays an important role in clinical outcome. Patients with WHO grade III meningioma had both poorer overall survival and relapse free survival rates than those with WHO grade II tumors, similar to articles by Palma *et al*.^28^. and Perry *et al*.^25^. The worst prognosis was attached to meningiomas with frank histological anaplasia, whether invasive or not^1^. Therefore, the 2007 revision identified brain invasion as the primary criterion for Grade II lesions, and a greater percentage of meningiomas have been classified as atypical over the last decade.

The location of atypical and malignant tumors significantly influences the RFS in the present study, in agreement with Kane *et al*.^5^. Dziuk *et al*. reported that the parasagittal-falcine lesions present with the highest recurrence rates through reviewed recurrent atypical and anaplastic tumors^29^. In our study, the RFS is decreased significantly in patients with parasagittal and posterior fossa tumors (HR = 2.2, p = 0.005). These tumors often were attached to or invade the sinus wall of the superior sagittal sinus or transverse sinus. This tumor location increases the difficulty of radical resection and may obscure residual tumor due to sinus enhancement, which may mislead the surgeon on the completeness of the tumor resection.

Embolization of meningioma prior to surgery has been performed for many years, although no dedicated studies assess its role in the treatment of atypical and malignant meningiomas. In our study, patients who underwent embolization had less average bleeding volumes during the operation. There was also a trend to a higher rate of GTR, which prolong RFS consequently. No patients presented complications following embolization, including contrast medium renal toxicity, stroke, carotid dissection, or acute deterioration caused by bleeding within a necrotic meningioma. The results suggested that atypical and malignant meningiomas respond positively to embolization before operation, especially those hypervascular tumors identified on image studies.

For benign meningioma, GTR is the standard surgical principle. Ayal *et al*. disclosed that the extent of resection is a powerful predictor of outcome for patients with atypical and malignant meningioma^30^. However, radical excision is complicated in some area like skull base because of many critical structures. Severe morbidity, even mortality may be the consequence of aggressive resection. In order to preserve neurological function, STR can be considered as an alternative surgical management. We previously showed that GTR increases progression free survival in patients with skull base atypical meningiomas^31^, consistent with previous findings by Yang *et al*.^32^, Goyal *et al*.^7^, and Zaher *et al*.^33^. Conversely, Rosenberg *et al*.^34^, Boskos *et al*.^35^, and Pasquier *et al*.^26^ found that the extent of resection was not a significant prognostic factor for OS or PFS in their RT-treated cohorts. In our study, GTR significantly prolonged RFS in atypical and malignant meningioma patients, in spite of no correlation with OS. Six patients (8.7%) who underwent GTR developed neurologic deficit like facial palsy or hemiparesis, while four patients (12.1%) with STR had facial palsy. The relatively low complication rate after GTR infers that extensive surgical strategy is able to prolong relapse free survival and cause no neurologic sequelae with modern microsurgical techniques.

The outcomes of patients who received adjuvant radiotherapy treatment following surgery for atypical meningioma are inconsistent. The latest systematic review revealed that adjuvant radiotherapy generally improves local control and OS in atypical and malignant meningiomas, although available data did not support this paradigm in the controversial subset of totally excised atypical meningiomas^4^. In our series, whether patient had adjuvant radiotherapy alone is not a prognostic factor to OS and RFS. In addition, adjuvant radiotherapy in patients who underwent GTR showed no significant association with RFS although GTR with adjuvant radiotherapy contributed to lower recurrent rate. However, radiation therapy was associated with decreased local recurrence among patients undergoing STR (but not GTR) by using a Kaplan-Meier analysis. These data suggest that adjuvant radiotherapy does not provide additional benefit for local tumor control in patients with complete tumor resection. Otherwise, STR combined with adjuvant radiotherapy reduced tumor progression with similar efficacy as GTR. Physicians should consider the difficulty of evaluating the extent of resection in posterior fossa and parasagittal tumors, and adjuvant radiotherapy can provide the benefits. We recommend adjuvant RT for all tumors that occupy these two locations.

The correlation between histological grading and Ki-67 antigen expression in meningioma is strong, and the antigen expression can be detected by the MIB-1 monoclonal antibody^36^. Although a poor prognosis may be associated with a high MIB-1 labeling index, significant overlap exists in the MIB-1 labeling ranges for benign, atypical, and anaplastic meningiomas^1^. It remains undetermined to the association of proliferation and clinical outcomes. Previous studies indicated that there is no correlation between two in all meningioma groups^37^,^38^. However, our analysis showed MIB-1 index is a significant prognostic factor for clinical outcome. We report that MIB-1 index >8% was significantly associated with worse OS and RFS in patients with atypical or malignant meningioma. The hazard ratio for OS and RFS are 19.31 (p = 0.005) and 2.89 (p = 0.001), respectively. This result agreed with a previous study that tumor proliferation elevation is correlated with decreased overall survival rate and higher tumor recurrent rate for both atypical and malignant meningiomas^39^.

## Patients and Methods

The records for all patients who were treated for atypical (WHO grade II) and malignant (WHO grade III) meningiomas between June 2001 and November 2009 at Chung Gang Memorial Hospital were retrospectively reviewed. This study was approved by the Chung Gang Memorial Hospital institutional review board, with approval number as 101–4601B. All methods were performed in accordance with the relevant guidelines and regulations.

Each patient’s clinical information was recorded as following: gender; age at diagnosis; tumor location; receipt of pre-operative embolization; extent of tumor resection; tumor pathology; progression or recurrence details; use of postoperative adjuvant radiotherapy; and the length and outcome at follow up. Karnofsky Performance Status (KPS) was determined from clinical notes. Atypical (WHO grade II) and malignant (WHO grade III) meningiomas were diagnosed based on histopathology with the latest 2007 WHO criteria. Older specimens were classified using current criteria retrospectively. Because the goal of this study was to analyze the clinical behavior of de novo atypical and malignant meningiomas, we excluded patients with confounding neurosurgical histories and patients with tumor recurrence after being treated previously for meningiomas. A total of 102 patients were included in this study.

### Preoperative Embolization

A neuro-radiologist performed a diagnostic cerebral angiography within three days of operation to assess for hypervascular tumor. Operative neurosurgeon discussed with neuro-radiologist before deciding whether to perform trans-arterial embolization (TAE). TAE procedure involved identifying the tumor’s main supplying artery, guiding a superselective cannulation into the feeding artery with a micro-catheter system, embolizing vessels with permanent particles, and checking for diminished contrast medium tumor stain.

### Surgery and Adjuvant Radiotherapy

All patients underwent a surgical resection after diagnosis of atypical and malignant meningioma. The degree of resection was determined using Simpson’s classification and based on the intraoperative impression of the surgeon and the radiographic evidence postoperatively. In our analysis, GTR was equivalent to Simpson Grades 1–3; subtotal removal(STR) was equivalent to Simpson Grade 4. GTR was achieved in 69 patients (67.6%), while 33 patients (32.4%) underwent STR. Radiotherapy (54–60 Gy, delivered in 27–30 fractions) was performed on 42 out of 102 (41.2%) patients following surgical intervention within 6 months of surgery (before any tumor relapse signs with radiographic or clinical evidence).

### Follow up evaluation

The mean follow-up time was 77.7 months (range: 5–192 months). We defined disease relapse as recurred tumors after GTR or enlarged residual tumor after STR. Gadolinium-enhanced MRI was used as radiological evidence in all cases assessment. Relapse free survival (RFS) was calculated from the day of GTR to the time of tumor recurrence, or from the day of STR to the time of tumor progression.

### Statistical Analysis

Survival and tumor relapse analysis was performed using Kaplan-Meier analysis, and comparisons between groups were performed using log-rank tests. The Cox model was used for continuous variables. A p value < 0.05 was considered statistically significant. Statistically significant risk factors after univariate analysis were subsequently entered into the multivariate analysis to identify risk factors for overall survival and relapse free survival. SPSS software (version 19; SPSS Inc. IBM Corporation) was used to perform statistical analysis.

## Limitations

The retrospective design of this study is susceptible to random bias. Although our series is one of the largest series that describes outcomes among patients with atypical and malignant meningiomas, the sample size was insufficiently powered to compare the relative impact of some variables in patients. Second, one single institute data are vulnerable to selection bias and dependent on the local surgical team. These findings may or may not reflect outcomes in meningiomas treated at other hospitals, as observed in the range of OS rates^4^. Finally, only the initial diagnosis of a tumor is included in our study, which may underestimate the percentage of WHO grade II and grade III meningiomas. Prior studies have demonstrated that around 14% to 29% of recurrent benign tumors will be classified as atypical or malignant meningiomas^1^,^40^.

## Conclusion

The present retrospective case series using the latest WHO grading system confirmed that malignant meningiomas contributed to poorer overall survival and relapse free survival than atypical lesions. Tumor embolization prior to surgery may be helpful in hyper-vascular lesions and may provide benefit for prognosis without eliciting additional complications. Total excision is recommended for both groups of meningiomas, if no severe complications are expected consequently. Adjuvant radiotherapy following surgical resection is advisable, especially for incompletely excised tumors or tumors located in the parasagittal area or posterior fossa. In addition, higher MIB-1 index (>8%) contributed to worse clinical outcomes. Multicenter, prospective studies are necessary to clarify management and more relevant prognostic factors in atypical and malignant meningiomas.

## Additional Information

**How to cite this article**: Wang, Y.-C. *et al*. Long Term Surgical Outcome and Prognostic Factors of Atypical and Malignant Meningiomas. *Sci. Rep.* **6**, 35743; doi: 10.1038/srep35743 (2016).

## Figures and Tables

**Table 1:** Patient Demographics and Tumor Characteristics.

	Number(%)	WHO Grade	
		II	III
All patients	102	86	16
Gender			
Male	45(44.1)	39	6
Female	57(55.9)	47	10
Age			
Mean ± SD	57.0 ± 16.7	55.5 ± 16.9	65.8 ± 13.0
KPS			
Mean ± SD	74.3 ± 8.3	74.2 ± 8.0	74.6 ± 10.0
Tumor Size (cm)			
Mean ± SD	5.2 ± 2.0	5.1 ± 2.1	5.5 ± 1.5
Location			
Covexity	33(32.4)	31	2
Parasagittal	32(31.4)	24	8
Skull base	29(28.4)	26	3
Posterior fossa	8(7.8)	5	3
Surgical Resection			
GTR	69(67.7)	57	12
STR	33(32.3)	29	4
Adjuvant Radiotherapy			
Yes	42(41.2)	32	10
No	60(58.8)	54	6
MIB-1 index			
Mean ± SD	8.6 ± 6.1	7.2 ± 3.9	16.1 ± 9.5
Recurrence or progression			
Yes	52 (51)	38	14
No	50 (49)	48	2

Abbreviations: WHO, World Health Organization; SD, Standard Deviation; GTR, Gross Total Resection; STR, Subtotal Removal.

**Table 2:** Factors Associated with Overall and Relapse Free Survival.

Factors	Overall Survival (P value)		Relapse Free Survival (P value)	
	Univariate Analysis	Multivariate Analysis	Univariate Analysis	Multivariate Analysis
Age	0.482	—	0.671	—
Sex	0.033*	0.049*	0.519	—
KPS	0.016*	0.006*	0.480	—
WHO grade	0.001*	0.039*	0.000*	0.042*
Tumor Diameter	0.883	—	0.413	—
Tumor Location	0.304	—	0.011*	0.122
Surgical Extent	0.284	—	0.010*	0.004*
Adjuvant RT	0.390	—	0.393	—
MIB Index	0.003*	0.272	0.003*	0.012*

^*^p < 0.05.

**Table 3:** Embolization of Meningiomas.

Preoperative Embolization	Patient number	Tumor size mean cm (SD)	Operative bleeding mean cc. (SD)	GTR number (%)
Yes	29	5.2 (1.9)	706 (217)	23(79.3)
No	73	5.3 (2.1)	1043 (303)	46(63.0)
